# Paraganglioma of the mediastinum: challenges in diagnosis and surgical management

**DOI:** 10.1186/1749-8090-5-19

**Published:** 2010-03-31

**Authors:** Ori Wald, Oz M Shapira, Aiman Murar, Uzi Izhar

**Affiliations:** 1 Department of Cardiothoracic Surgery, Hadassah University Hospital, Jerusalem, 91120 P.O.B 12000, Israel

## Abstract

Mediastinal paraganglioms are rare, highly vascularized tumors arising from chromaffin tissue located in the para-aortic ganglia. Tumors tend to invade bordering structures and may also form metastasis. Up to 50% of patients are asymptomatic and diagnosis is incidental. Presenting symptoms are related to catecholamine hypersecretion or to a mass effect. Complete surgical resection remains the standard of care due to malignant potential of the tumor and poor response to chemotherapy or radiation. Strategic location of the tumor in proximity to great vessels, trachea, and recurrent laryngeal nerve poses challenge for the surgeon. We report a case of a 59-year old asymptomatic female who was incidentally diagnosed with a middle mediastinal mass on a positron-emission tomography (PET-CT) scan performed as part of breast cancer surveillance. Complete resection of the tumor was achieved using cardiopulmonary bypass. The patient recovered uneventfully and in a ten-month follow up there is no evidence of recurrence.

## Introduction

Ninety percent of chromaffin-cell-originating tumors are located in the adrenal gland and termed pheochromocytomas. The remaining ten percent are extra adrenal and are termed paragangliomas. Paragangliomas appear in the abdomen, pelvis, neck and mediastimun. Mediastinal paraganglioma originate from para-aortic (middle mediatsinum) and para-vertebral (posterior mediatsinum) sympathetic chain ganglia [1,2]. Similar to pheochromocytoma, paraganglioma tumors may secrete catecholamines, however in majority of cases they are non-functional. Up to 50% of patients are asymptomatic and the diagnosis is incidental [1]. Clinical symptoms may be related to catecholamine hypersecretion (hypertention/hyperhydrosis) or to a mass effect resulting in complains of hoarseness, dysphagia, shortness of breath and chest pain [3].

## Case

**Figure 1:**
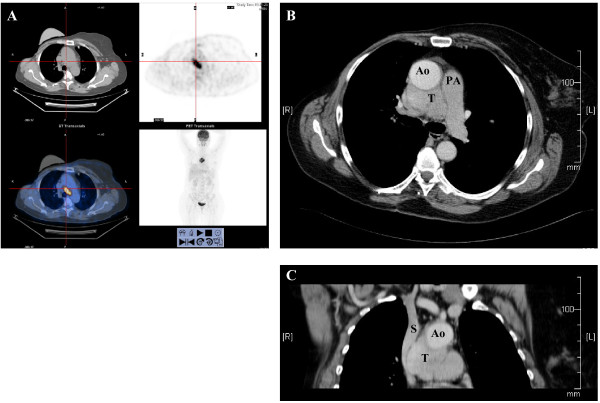
**A - PET CT scan image showing a middle mediastinal mass of 5 cm in diameter with an SUV of 20**. B, C - Axial and coronal CT scan images showing the tumor (T) adjacent to the aorta (Ao) and pulmonary artery (PA). The compressed superior vena cava is seen in coronal view (SVC).

A 54-year-old woman was referred for resection of an asymptomatic middle mediastinal mass. Her past medical history was remarkable for right breast carcinoma treated with lumpectomy, axillary lymph node dissection, adjuvant chemotherapy and irradiation sixteen years prior to this admission. Six years later a right para-vertebral desmoid tumor was completely resected. One year ago a second right breast carcinoma was diagnosed and completion mastectomy was performed. A positron emission tomography - computerized tomography (PET-CT scan) performed prior to her admission as part of her oncological follow-up revealed a 5-cm middle mediastinal mass with a standardized uptake value (SUV) of 20 (Fig 1A). With a differential diagnosis of an infectious process a course of antibiotics was administered without response. Repeat computerized tomography of the chest (CT scan) demonstrated a 5-cm mass, located between the aorta and the superior vena cava, compressing the right pulmonary artery, and adherent to the anterior tracheal wall (Fig 1B, 1C). Trans-bronchial biopsy was suggestive of a typical carcinoid tumor and the patient was referred to surgery.

Upon admission, the patient denied any symptoms related neither to catecholamine hyper-secretion nor to carcinoid syndrome. Physical examination and routine laboratory results were unremarkable.

**Figure 2:**
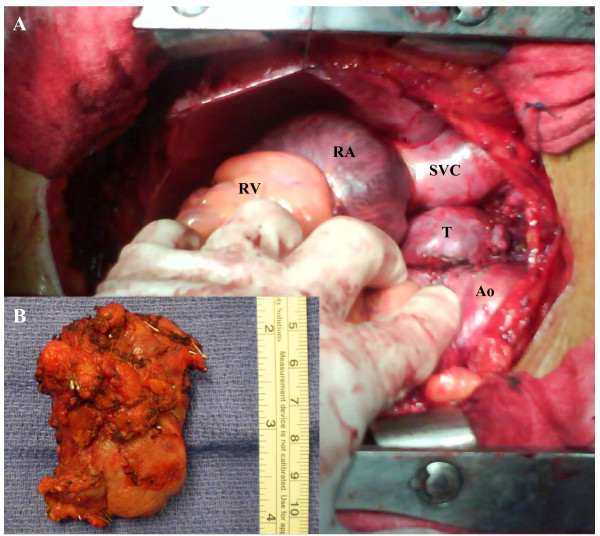
**A - Intraoperative view: the tumor (T) is seen between the aorta (Ao) and superior vena cava (S)**. Right atrium (RA) Right ventricle (RV). B - resected tumor is shown.

The operation was performed via a median sternotomy. A soft, highly vascularized, 1.5 × 2.5 × 5.5 cm mass located between the aorta, the superior vena cava and the right atrium was identified. The tumor compressed the right pulmonary artery, and was densely adherent to the aorta and the anterior wall of the trachea with a very rich network of small blood vessels (Fig 2). Frozen section confirmed the diagnosis of a neuroendocrine tumor with a low mitotic index. Because of these findings we elected to remove the tumor using cardiopulmonary bypass to allow complete and safe resection. We performed a right femoral artery and the right atrium cannulaion, allowing manipulation and possible excision of the ascending aorta. The mass was completely resected. Postoperative course was remarkable for a transient left vocal cord paralysis. Final pathological examination demonstrated the characteristics architecture of paraganglioma. The tumor cells stained positive for synaptophysin chromogranin and sustentacular cells stained positive to S-100. Proliferation index was 5%. Cytology result of pericardial effusion did not show malignant cells.

The patient has been followed-up since surgery. Currently she is asymptomatic. Both chest MRI and a whole body FDG PET-CT scan, performed 10 months postoperative, did not show evidence of recurrence.

## Comment

The diagnosis of a paraganglioma is based on clinical symptoms, imaging tests and urinary essays of catecholamine metabolites. Patients with inactive tumors may present with large tumors that compress or invade neighboring structures [1,4].

Paragangliomas have typical imaging characteristics on CT and magnetic resonance imaging (MRI) scans. Paragnagliomas are usually located in the bifurcation of great vessels and show intense and homogeneous enhancement except for necrotic areas that enhanced poorly. MRI images show intermediate signal intensity on T1-weighted images and high signal intensity on T2-weighted images [2]. ^123^I-MIBG (^123^I-metaiodobenzylguanidine) scintigraphy and PET-CT scan with 18-FDG (18-fluorodeoxyglucose) are used for localization and staging of these tumors when appropriate. CT-guided needle biopsy is not mandatory and, in fact, can be hazardous due to the proximity of the tumor to the great vessels and its intense vascularity. Our patient's history suggested a differential diagnosis for the current mass of either tumor recurrence or a third primary tumor. Therefore preoperative tissue diagnosis was indicated. Needle-biopsy-based tissue diagnosis of paraganglioma is difficult and complete pathological examination including tumor morphology and structure and specific stains are necessary to achieve accurate diagnosis.

Complete resection is the standard of care of paraganglioma, affording the patient with the best chance of cure since these tumors are relatively resistant to chemotherapy and irradiation [5]. The highly vascular nature of these tumors and strategic anatomical locations make complete resection demanding, and often mandates the use of median sternotomy and cardiopulmonary bypass [6,7]. In most cases these tumors can be removed in a single-stage operation. A recent report described a two-stage approach for resection of a paraganglioma invading the pulmonary artery and ascending aorta [8].

The two major concerns involving resection of mediastinal paragngliomas include intraoperative bleeding and catecholamine crises in patients with metabolically active tumors. The highly vascular nature of the tumor, the proximity and invasion to the great arteries and the systemic anticoagulation necessary for cardiopulmonary bypass - all contribute to a high risk of bleeding. [9]. Hormonal-related crises are uncommon but are associated with significant morbidity and mortality [1,10]. Meticulous surgical technique and tight preoperative blood pressure control are the key steps in prevention and management of these complication [7].

Prognosis after complete resection is favorable. Lamy et al reported a follow up of 79 patients with middle mediastinal paragangliomas over a period of 180 months. Among these patients overall survival was 62.0%, mean survival time was 98.2 +/- 11.7 months (mean +/- standard error). For patient undergoing complete resection survival was 84.6%, mean survival time was 125.7 +/- 18.7 months (mean +/- standard error). For patient undergoing incomplete resection survival was 50.0% mean survival time was 71.5 +/- 13.8 months (mean +/- standard error) [5]. In a literature review of extraadrenal chromaffin cells tumors Erickson et al has reported a surgical cure rate of 69% [10].

In summary, paragnagliomas should be included in the differential diagnosis of a middle mediastinal mass. Complete surgical resection remains the standard of care and is associated with excellent survival. Life-long surveillance for local recurrence and metastatic spread is mandatory.

## Competing interests

The authors declare that they have no competing interests.

## Authors' contributions

OW reviewed the literature and wrote the manuscript, UI and OMS operated on the patient and edited the manuscript. IM took part in operation, and prepared the figures. All authors read and approved the final manuscript.
